# Factors affecting unusual postoperative vertical downward movement of mandible after surgery-first approach using intraoral vertical ramus osteotomy

**DOI:** 10.1186/s12903-024-05021-y

**Published:** 2024-10-10

**Authors:** Soo-Hyun Nam, Jung-Yul Cha, Kee-Joon Lee, Sang-Hwy Lee, Sung-Hwan Choi, Hyung-Seog Yu

**Affiliations:** 1 Department of Orthodontics, Institute of Craniofacial Deformity, Yonsei University College of Dentistry, 50-1 Yonsei-Ro, Seodaemun-Gu, Seoul, 03722 Republic of Korea; 2 Department of Oral and Maxillofacial Surgery, Oral Science Research Center, Yonsei University College of Dentistry, 50-1 Yonsei-Ro, Seodaemun-Gu, Seoul, 03722 Republic of Korea

**Keywords:** Surgery-first, POGS, IVRO, Stability

## Abstract

**Background:**

In very rare cases, patients who have undergone surgery-first approach with intraoral vertical ramus osteotomy (IVRO) exhibit unusual downward movements of mandible even up to 1-year post surgery, which makes it difficult for orthodontists to stabilize the occlusion during the postoperative orthodontic period. The aim of this study was to identify factors affecting the unusual downward movement of the mandible 1-year after the surgery-first approach using IVRO, while focusing on cephalometric values.

**Methods:**

This retrospective cohort study sample was divided into two groups based on the amount of vertical movement of the B-point 1-year post surgery (Group S, predictable upward movement; Group U, unpredictable downward movement greater than 2 mm). To evaluate cephalometric changes between the two groups, cephalograms were obtained before surgery, 1 day after surgery, 1 month after surgery, and 1 year after surgery. The data were analyzed using the independent *t-*test, Mann–Whitney *U* test with Bonferroni correction, Pearson correlation analysis, and multiple regression analysis.

**Results:**

At the initial examination, Group U showed a shallower anterior overbite. The vertical surgical change in the B-point was statistically different between the two groups (*p* < 0.001), indicating that group U exhibited more upward movement of the mandible during surgery. Group U showed significant downward movement of the mandible 1 month after surgery, and this finding persisted until 1 year postoperatively. Clockwise rotation of the mandible was also observed. Surgical vertical movement of the B-point showed a strong correlation with postoperative vertical movement of mandible (*r* = -0.674; *p* < 0.001) along a linear relationship, indicating that the amount of postoperative vertical downward movement of the mandible increased as the amount of surgical upward movement of the B-point increased (R^2^ = 0.449; *p* < 0.001).

**Conclusions:**

This study revealed that unusual downward movement of the mandible after a surgery-first approach using IVRO is correlated with the amount of upward movement during the surgery. When planning surgery, in cases in which a significant upward movement of the mandible is anticipated, orthodontists should prepare for the possibility of subsequent unusual downward movement and a tendency for the anterior overjet to decrease during the postoperative orthodontic period.

## Background

Skeletal Class III malocclusion has a prevalence of 4 to 14% among Asian people, which is higher when compared to the corresponding prevalence among other races, and the majority of these cases show an overdeveloped mandible [1–3]. To treat patients with severe skeletal Class III, for which orthodontic treatment alone cannot achieve normal occlusion, bimaxillary surgery, including maxillary Le-fort I osteotomy and mandibular setback, is often performed [4, 5]. Recently, the development of TADs (temporary anchorage devices) has expanded the scope of orthodontic treatment [6] and led to an increase in the surgery-first approach that bypasses the preoperative orthodontic treatment. The surgery-first approach is increasingly being performed because it provides immediate aesthetic improvements to patients, high patient satisfaction, and more efficient tooth movement postoperatively, particularly in asymmetric cases, which shortens the total treatment duration [7–10].

Numerous studies have examined stability after a surgery-first approach. In their systematic review, Soverina et al. [11] reported that the surgery-first approach shows stability and predictability comparable to conventional surgery. From the perspective of vertical stability, Choi et al. [12, 13], Ann et al. [14], Kim et al. [15], and Jeong et al. [16] reported that, in a surgery-first approach using IVRO (Intraoral Vertical Ramus Osteotomy), the mandible primarily moves upward 1-year post surgery. In other studies, Yoshioka et al. [17] and Nihara et al. [18] reported that clockwise rotation of the mandible occurred within 3 months of IVRO, and that such rotation is likely attributable to condylar sag and muscular pull occurring immediately after surgery. However, after a surgery-first approach, premature occlusal contacts disappear during postoperative orthodontic treatment, and the masticatory muscles are rehabilitated, and these outcomes contribute to upward movement of the mandible 1-year post surgery [12].

As was expected, almost all patients who have undergone a surgery-first approach using IVRO exhibited upward movement of the mandible 1-year post surgery. However, in very rare cases, patients exhibited unusual downward movements of the mandible even up to 1-year post surgery, and these movements make it difficult for orthodontists to stabilize occlusion during postoperative orthodontic treatment. To our knowledge, there have been few studies investigating the factors that affect the clinically challenging vertical downward movement occurring in the mandible after a surgery-first approach using IVRO.

Conventionally, when surgical healing is complete, jaw movements exceeding a distance of 2 mm from the position of the time of surgery are considered clinically significant [19]. Therefore, the aim of this study was to identify factors affecting the unusual downward movement of the mandible exceeding 2 mm 1-year after the surgery-first approach using IVRO, while focusing specifically on cephalometric values. This study also investigated the correlation between the considered factors and postoperative vertical downward movement of the mandible post-surgery.

## Methods

### Study design and patients

This retrospective cohort study included adult patients who were diagnosed with skeletal Class III malocclusion and who were treated with bimaxillary surgery using IVRO in a surgery-first approach by the same surgeon between 2010 and 2020 at the Department of Oral and Maxillofacial Surgery, Yonsei Dental Hospital, Seoul, Republic of Korea.

The inclusion criteria for this study were as follows: 1) Age 18 years or older; 2) Skeletal Class III malocclusion with an ANB angle (the angle formed by point A, the nasion, and point B) < 0º; 3) Requirement of bimaxillary surgery (one-piece Le Fort I osteotomy and bilateral IVRO with or without genioplasty); 4) Surgery-first approach without preoperative orthodontic treatment; 5) Maxillomandibular intermolar width difference greater than 6 mm [20]; 6) Complete series of lateral and posterior-anterior cephalogram.

The exclusion criteria were as follows: 1) History of previous orthognathic surgery; 2) Requirement of single-jaw surgery; 3) Requirement of conventional surgery with preoperative orthodontic treatment; 4) Congenital craniofacial anomalies; 5) Temporomandibular joint or muscle disorder.

**Fig. 1:**
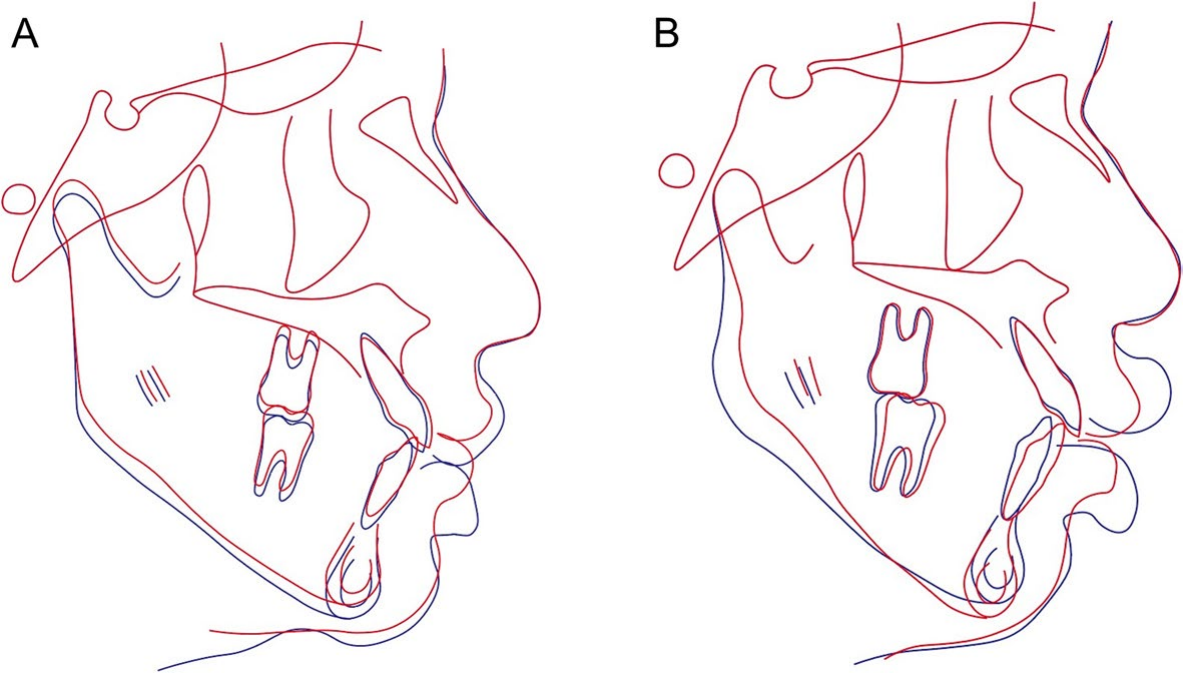
Movement of the mandible 1-year post surgery. **A**, demonstrates predictable upward movement of the mandible; **B**, shows unexpected downward movement of the mandible; Blue line: 1 day after surgery; Red line: 1 year after surgery

Patients who showed an upward movement of the mandible 1-year post surgery were assigned to Group S (Fig. 1, A). As previously mentioned, a jaw movement greater than 2 mm, when compared from the position at the time of surgery, is considered clinically significant once surgical healing is complete. [19]; accordingly, this study included patients whose B-point moved downward more than 2 mm 1-year after surgery as Group U (Fig. 1, B). Hereafter, this study defines 'vertical instability' or being 'vertically unstable' as instances in which the B-point of the mandible moved downward more than 2 mm 1-year post surgery. The procedures in this study followed the guidelines of the Declaration of Helsinki and were approved by the Institutional Review Board of Yonsei Dental Hospital (2–2024-0020).

### Surgical and orthognathic treatment

Five weeks before surgery, surgical archwires made of 0.017 × 0.025-inch stainless steel were directly bonded to the teeth for intermaxillary fixation (IMF) after surgery. No orthodontic forces were applied before surgery. All orthodontic treatments were done by the same orthodontist at the Department of Orthodontics, Yonsei Dental Hospital.

All surgeries were performed by the same surgeon, and all patients were treated according to the same surgical and postoperative protocol. After the maxillary Le Fort I osteotomy, four prefabricated rigid L-shaped titanium plates were fixed with screws in both the canine fossa and the zygomatic buttress. The mandible was setback bilaterally using IVRO, and no fixation was performed between the proximal and distal segments. The osteotomy line of the mandible extended from the mandibular angle to the sigmoid notch. Post-surgery, IMF was implemented for one week, followed by active physiotherapy (PT) three times a day, comprising of 30–40 repetitions of mouth opening to guide the mandible into the planned position. For eight weeks post-surgery, patients were instructed to wear bilateral intermaxillary elastics throughout the day while changing them once daily. Postoperative PT was conducted with the goal of achieving a maximum mouth opening of over 40 mm, and it included a cooperation assessment that was administered during outpatient visits occurring every week for about eight weeks. The final wafer was removed from the patient at three weeks post-surgery, but they were instructed to wear it with elastics at night. Eight weeks after the surgery, the patients were referred to the orthodontic department to begin postoperative orthodontic treatment.

### Data collection methods

Skeletal and dental changes were evaluated and compared before and after the surgery using lateral cephalograms and posterior-anterior cephalograms. Cephalograms were taken before surgery (T1), one day after surgery (T2), one month after surgery (T3), and 1 year after surgery (T4). Surgical changes were assessed from T2-T1, immediate postoperative changes were assessed from T3-T2, and final postoperative changes were assessed from T4-T2. The lateral cephalogram and posterior-anterior cephalogram measurements were performed using V-ceph 5.5 (Osstem, Seoul, Korea), and changes were assessed by superimposing these measurements on the sella-nasion (SN) plane in the lateral cephalogram.

Measurements in the lateral cephalogram were made according to an x–y coordinate system where the x-axis was defined as a line passing through N and forming 7º upwards from the SN plane, while the y-axis was defined as a line passing through S and perpendicular to the x-axis. The locations of the measurement points were determined by their distances from the x- and y-axes. In the posterior-anterior cephalogram, the amount of mandibular menton deviation was evaluated based on a horizontal reference line passing through bilateral Z-points and a vertical reference line perpendicular to the horizontal reference line passing through the crista galli. The distance of the menton from the vertical reference line was measured.

**Fig. 2:**
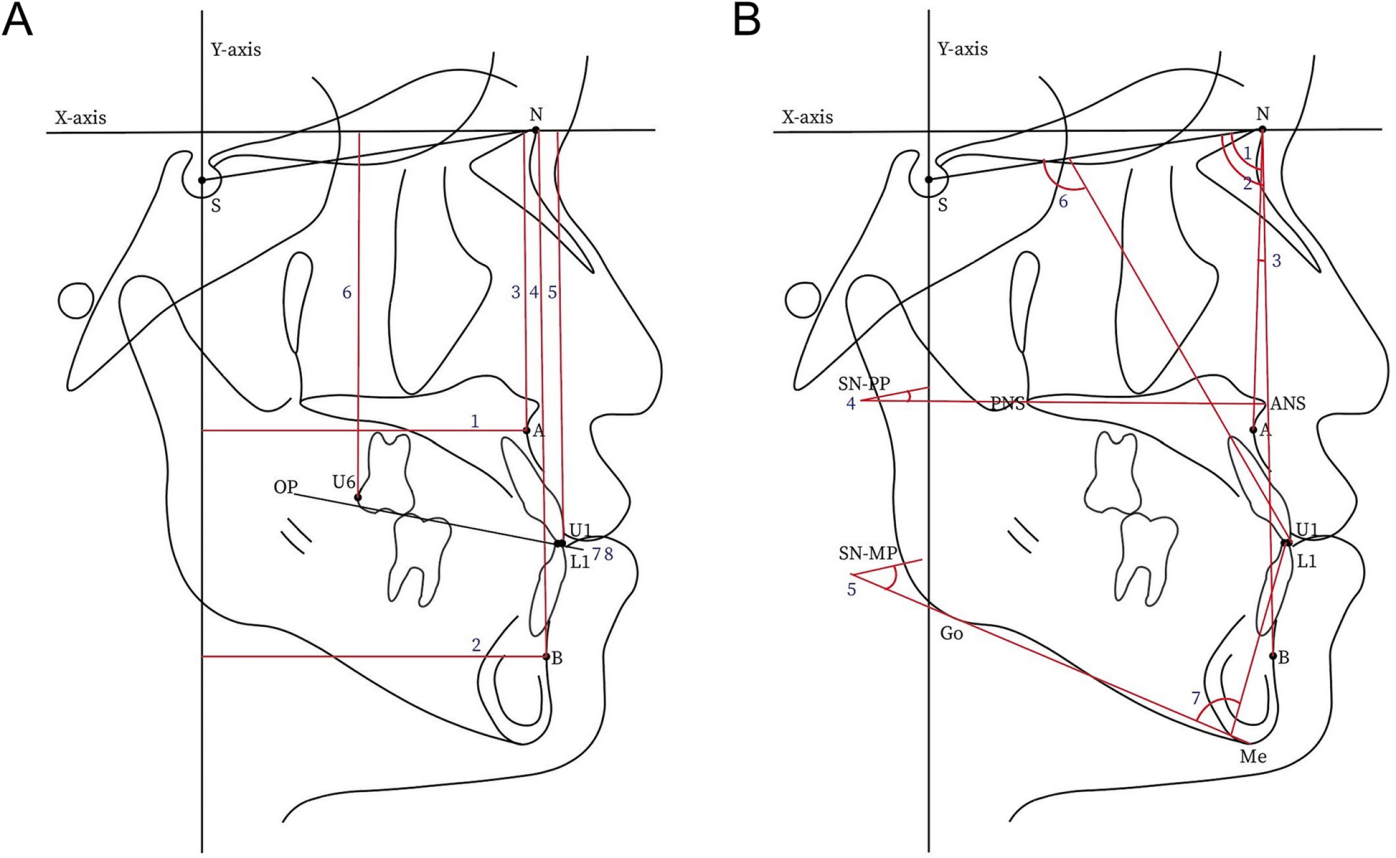
**A**, Cephalometric linear measurements. 1, A[X], horizontal position of point A; 2, B[X], horizontal position of point B; 3, A[Y], vertical position of point A; 4, B[Y], vertical position of point B; 5, U1[Y], vertical position of upper central incisor; 6, U6[Y], vertical position of upper first molar; 7, OJ, Subtract distance of L1 from U1 parallel to OP; 8, OB, Subtract distance of L1 from U1 perpendicular to OP; A, point A; B, point B; S, sella; N, nasion; U1, tip of upper central incisor; U6, tip of distal cusp of upper first molar; OP, occlusal plane; X-axis, defined by origin of nasion and forming 7º angle upward from SN plane; Y-axis, defined as a line perpendicular to X-axis and passing through sella. **B**, Cephalometric angular measurements. 1, SNA, angle of lines formed by S, N, and point A; 2, SNB, angle of lines formed by S, N, and point B; 3, ANB, angle of lines formed by point A, N, and point B; 4, SN-PP, angle between the SN plane and the palatal plane; 5, SN-MP, angle between the SN plane and the mandibular plane; 6, U1-SN, angle of the SN plane to the upper incisor; 7, IMPA, angle of the mandibular plane to the lower incisor; A, point A; B, point B; S, sella; N, nasion; U1, tip of upper central incisor; L1, tip of lower central incisor; ANS, anterior nasal spine; PNS, posterior nasal spine; Go, gonion; Me, menton

This study identified nine linear measurements and seven angular cephalometric measurements. The nine linear measurements included the horizontal distance from point A and point B to y-axis; the vertical distances from each of point A, point B, the tip of the upper central incisor (U1), and the tip of the distal cusp of the upper first molar (U6) to the x-axis; anterior overjet (OJ) and overbite (OB, Fig. 2, A); and amount of menton deviation.

The seven angular measurements included the angle of the lines formed by S, N, and point A (SNA); the angle of the lines formed by S, N, and point B (SNB); the angle of the lines formed by point A, N, and point B (ANB); the angle between the SN plane and the palatal plane (SN-PP); the angle between the SN plane and the mandibular plane (SN-MP); the angle of the SN plane to the upper incisor (U1-SN); and the angle of the mandibular plane to the lower incisor (IMPA, Fig. 2, B).

### Reliability

Reproducibility was evaluated by comparing the measurements obtained from initial examinations with those obtained from repeated examinations. All measurements were repeated by the same examiner at 1-week intervals. The method error was calculated using the intraclass correlation coefficient, which was between 0.85–0.90 for all linear and angular measurements in this study.

### Statistical analysis

All statistical analyses were performed using IBM SPSS 26.0 for Windows (IBM Korea Inc., Seoul, Korea). The minimum sample size for detecting differences in cephalometric changes at each time point between groups using the independent *t*-test was calculated based on the previous study by Kwon et al. [21]: Using G*Power 3 (Düsseldorf, Germany) with targets of a significance level of a *p* value less than 0.05, a power of 80%, and an effect size of 1.26, the minimum sample size was confirmed as 8. The Shapiro–Wilk test was used to verify the normality of the data distribution. The values included in the sample characteristics were presented in the forms of means and standard deviations (SD). The Fisher's exact test and independent *t-* test were used for comparisons of the sample characteristics between groups. The independent *t*-test or Mann–Whitney *U* test with Bonferroni correction was used to compare the initial condition (T1) between the two groups, as well as the surgical change (T2-T1), immediate postoperative change (T3-T2), and final postoperative change (T4-T2). Changes over time within each group were analyzed using either the paired *t*-test or the Wilcoxon signed rank test with Bonferroni correction. A *p* value less than 0.008 (= 0.05/6) was considered to represent a statistically significant difference.

Factors influencing the vertical downward movement of the mandible 1 month and 1 year after surgery were analyzed separately. To explore factors that might affect downward movement of the mandible post-surgery, Pearson correlation analysis was conducted. In assessing the degree of correlation, an *r* value greater than 0.40 was considered to indicate a moderate-to-strong correlation, while an *r* value less than 0.40 indicated a weak correlation [22]. Multiple regression analysis was performed along with the stepwise method to determine the association between factors, with the results showing a strong association and downward movement of the mandible post-surgery. A *p* value less than 0.05 was interpreted as being statistically significant.

## Results

There were total of 91 patients who underwent surgery-first approach, and 82 of them met the inclusion criteria. Among these, twenty-four patients (Group S, 11 men and 13 women; mean age, 20.9 ± 2.9 y, Table 1) showed a vertical upward movement of the mandible 1-year post surgery. Ten patients (Group U, 7 men and 3 women; mean age, 20.5 ± 1.8 y, Table 1) exhibited a vertical downward movement of the mandible exceeding 2 mm 1-year post surgery (Fig. 1, B).

**Table 1:** Sample Characteristics (*N* = 34)

Variable	Group S	Group U	*p* value
	(*n* = 24)	(*n* = 10)	
Sex, *n* (%)			0.270 ^a^
Men	11 (45.8)	7 (70.0)	
Women	13 (54.2)	3 (30.0)	
Age (y)			
Mean ± SD	20.9 ± 2.9	20.5 ± 1.8	0.926 ^b^
Postoperative treatment period (m)			
Mean ± SD	12.8 ± 7.9	13.1 ± 4.5	0.322 ^b^

*Abbreviations: Group S* stable group, *Group U* unstable group

^a^The *p* value was calculated using the Fisher exact *t*-test

^b^The *p* value was calculated using the independent *t*-test

There were no significant between-group differences in the sample characteristics in terms of sex, age, or postoperative treatment period. The mean vertical movement of the B-point 1-year post surgery was 1.5 mm (SD, 1.2 mm) upward in group S and 4.7 mm (SD, 1.9 mm) downward in group U. There was a significant difference in the vertical movement of B-point 1-year post surgery between the two groups (*p* < 0.001).

### Observation at the initial examination

Meaningful between-group cephalometric differences (defined as *p* < 0.05/6) were observed in OB at the initial examination (Table 2): The mean OBs were 1.3 mm (SD, 2.8 mm) in group S and -1.6 mm (SD, 2.5 mm) in group U (*p* = 0.007), indicating that group U had a shallower anterior overbite.

**Table 2:** Comparison of cephalometric measurements at initial examination between groups

T1	Group S	Group U	*p* value
Linear measurements (mm)			
A[X]	67.9 ± 5.6	70.6 ± 4.0	0.169
B[X]	71.4 ± 8.0	73.9 ± 9.4	0.427
A[Y]	67.5 ± 4.0	66.8 ± 3.4	0.642
B[Y]	115.1 ± 7.7	122.5 ± 7.2	0.014
U1[Y]	92.4 ± 5.5	93.4 ± 5.0	0.614
U6[Y]	81.5 ± 2.2	84.0 ± 5.8	0.219
OJ	-2.0 ± 3.3	-2.3 ± 4.0	0.826
OB	1.3 ± 2.8	-1.6 ± 2.5	0.007†
Menton deviation	5.1 ± 3.9	4.9 ± 3.9	0.904
Angular measurements (º)			
SNA	79.8 ± 4.5	81.7 ± 4.3	0.267
SNB	82.8 ± 3.3	83.8 ± 5.0	0.521
ANB	-3.0 ± 3.5	-2.1 ± 2.3	0.441
SN-PP	10.4 ± 3.3	8.2 ± 2.7	0.068
SN-MP	34.4 ± 6.2	37.5 ± 3.5	0.137
U1-SN	108.6 ± 6.8	113.6 ± 7.2	0.062
IMPA	80.8 ± 8.6	83.7 ± 6.4	0.347

Data are presented as mean ± deviation. Group comparisons were tested using the independent *t*-test or Mann–Whitney *U* test with Bonferroni correction

*Abbreviations:* Group S, stable group; Group U, unstable group. A[X], horizontal position of point A; B[X], horizontal position of point B; A[Y], vertical position of point A; B[Y], vertical position of point B; U1[Y], vertical position of upper central incisor; U6[Y], vertical position of upper first molar; OJ, Subtract distance of L1 from U1 parallel to OP; OB, Subtract distance of L1 from U1 perpendicular to OP; Asymmetry, amount of menton deviation; SNA, angle of lines formed by the S,N, and point A; SNB, angle of lines formed by the S.N, and point B; ANB, angle of lines formed by point A, the N, and point B; SN-PP, angle between the SN plane and the palatal plane; SN-MP, angle between the SN plane and the mandibular plane; U1-SN, angle of the SN plane to the upper incisor; IMPA, angle of the mandibular plane to the lower incisor; T1. 1 month before surgery

^†^*p* < 0.05/6, significant difference between groups by independent *t*-test or Mann-Whiteny *U* test

^‡^*p* < 0.001, significant difference between groups by independent *t*-test or Mann-Whiteny *U* test

### Observation 1 day after surgery

The mean surgical movements at point A were 0.1 mm (SD, 3.1 mm) posteriorly and 0.8 mm (SD, 2.6 mm) superiorly in group S, while the corresponding values were 2.3 mm (SD, 3.1 mm) posteriorly and 1.7 mm superiorly (SD, 2.4 mm) in group U (Table 3). The differences in the horizontal and vertical surgical change of point A between the two groups were not statistically significant (*p* > 0.05/6).

**Table 3:** Comparison of surgical changes in cephalometric measurements between groups

T2-T1	Group S	Group U	*p* value
Linear measurements (mm)			
A[X]	-0.1 ± 3.1	-2.3 ± 3.1	0.066
B[X]	-9.3 ± 4.9**	-10.6 ± 6.5*	0.525
A[Y]	-0.8 ± 2.6	-1.7 ± 2.4	0.353
B[Y]	0.9 ± 3.8	-4.6 ± 3.3*	0.000‡
U1[Y]	0.5 ± 2.2	-1.0 ± 2.0	0.071
U6[Y]	-3.1 ± 2.2**	-3.8 ± 3.2*	0.461
OJ	6.2 ± 3.9**	7.0 ± 3.9**	0.583
OB	-0.5 ± 2.4	1.7 ± 2.5	0.025
Menton deviation	4.8 ± 3.9**	5.1 ± 3.6*	0.865
Angular measurements (º)			
SNA	-0.1 ± 3.0	-2.2 ± 3.1	0.083
SNB	-4.5 ± 2.6**	-5.1 ± 3.0*	0.623
ANB	4.4 ± 3.5**	2.9 ± 3.2*	0.244
SN-PP	4.4 ± 3.4**	3.7 ± 2.9*	0.601
SN-MP	4.0 ± 3.8**	0.7 ± 4.0	0.031
U1-SN	-3.6 ± 5.3*	-2.7 ± 6.7	0.678
IMPA	1.7 ± 3.0*	0.5 ± 2.4	0.264

Data are presented as mean ± deviation. Positive and negative values indicate anterior and posterior horizontal changes, respectively; inferior and superior vertical changes, respectively; or increased or decreased dimensional changes, respectively. Group comparisons were tested with independent *t*-test or Mann–Whitney *U* test with Bonferroni correction

*Abbreviations:* Group S, stable group; Group U, unstable group. A[X], horizontal position of point A; B[X], horizontal position of point B; A[Y], vertical position of point A; B[Y], vertical position of point B; U1[Y], vertical position of upper central incisor; U6[Y], vertical position of upper first molar; OJ, Subtract distance of L1 from U1 parallel to OP; OB, Subtract distance of L1 from U1 perpendicular to OP; Asymmetry, amount of menton deviation; SNA, angle of lines formed by the S,N, and point A; SNB, angle of lines formed by the S.N, and point B; ANB, angle of lines formed by point A, the N, and point B; SN-PP, angle between the SN plane and the palatal plane; SN-MP, angle between the SN plane and the mandibular plane; U1-SN, angle of the SN plane to the upper incisor; IMPA, angle of the mandibular plane to the lower incisor; T1, 1 month before surgery; T2, 1 day after surgery

^*^p < 0.05/6, significant difference between T1 and T2 by paired *t*-test or Wilcoxon signed rank test

^**^p < 0.001, significant difference between T1 and T2 by paired *t*-test or Wilcoxon signed rank test

^†^*p* < 0.05/6, significant difference between groups by independent *t*-test or Mann-Whiteny *U* test

^‡^*p* < 0.001, significant difference between groups by independent *t*-test or Mann-Whiteny *U* test

The mean surgical movement at point B was 9.3 mm (SD, 4.9 mm) posteriorly (*p* < 0.001) and 0.9 mm (SD, 3.8 mm) inferiorly in group S, while the corresponding values were 10.6 mm (SD, 6.5 mm) posteriorly (*p* < 0.05/6) and 4.6 mm (SD, 3.3 mm) superiorly (*p* < 0.05/6) in group U (Table 3). There was a statistically significant difference in the vertical surgical change of point B between the two groups (*p* < 0.001), indicating that group U had more upward movement of the mandible compared to group S during surgery.

### Observation 1 month after surgery

At 1 month after surgery, point A was found to moved 0.3 mm (SD, 1.2 mm) anteriorly and 0.1 mm (SD, 2.3 mm) superiorly in group S, while it was found to move 0.3 mm (SD, 1.3 mm) anteriorly and 0.1 mm (SD, 1.7 mm) superiorly in group U (Table 4). These changes did not differ significantly between the two groups. However, point B moved 1.8 mm (SD, 2.5 mm) posteriorly (*p* < 0.05/6) and 0.3 mm (SD, 2.0 mm) inferiorly in group S, while it moved 1.8 mm (SD, 2.1 mm) posteriorly and 2.8 mm (SD, 0.9 mm) inferiorly (*p* < 0.001) in group U (Table 4). The vertical change of the B-point 1-month post-surgery differed significantly between the groups (*p* < 0.001), indicating that immediate downward movement of the mandible occurred after surgery in group U. No meaningful between-group differences were observed for the angular measurements.

**Table 4:** Comparison of immediate postoperative changes in cephalometric measurements between groups

T3-T2	Group S	Group U	*p* value
Linear measurements (mm)			
A[X]	0.3 ± 1.2	0.3 ± 1.3	0.929
B[X]	-1.8 ± 2.5*	-1.8 ± 2.1	0.940
A[Y]	-0.1 ± 2.3	-0.1 ± 1.7	0.944
B[Y]	0.3 ± 2.0	2.8 ± 0.9**	0.000‡
U1[Y]	0.2 ± 1.2	0.6 ± 0.7	0.287
U6[Y]	-0.6 ± 1.1	-0.9 ± 1.7	0.616
OJ	0.2 ± 1.0	-0.7 ± 1.4	0.046
OB	-0.3 ± 1.3	-0.6 ± 1.3	0.556
Menton deviation	0.5 ± 0.5	0.4 ± 0.2	0.363
Angular measurements (º)			
SNA	0.5 ± 1.4	0.6 ± 1.4	0.912
SNB	-0.7 ± 1.4	-0.7 ± 1.1	0.931
ANB	1.2 ± 1.2**	1.3 ± 1.7	0.845
SN-PP	-0.2 ± 3.0	0.6 ± 2.1	0.454
SN-MP	2.6 ± 1.6**	4.1 ± 1.8**	0.026
U1-SN	-1.1 ± 3.2	-2.4 ± 3.9	0.327
IMPA	-1.0 ± 3.6	-2.1 ± 2.7	0.413

Data are presented as mean ± deviation. Positive and negative values indicate anterior and posterior horizontal changes, respectively; inferior and superior vertical changes, respectively; or increased or decreased dimensional changes, respectively. Group comparisons were tested with independent *t*-test or Mann–Whitney *U* test with Bonferroni correction

*Abbreviations: Group S* stable group, *Group U* unstable group. A[X], horizontal position of point A, B[X], horizontal position of point B, A[Y], vertical position of point A, B[Y], vertical position of point B, U1[Y], vertical position of upper central incisor, U6[Y], vertical position of upper first molar, OJ, Subtract distance of L1 from U1 parallel to OP; OB, Subtract distance of L1 from U1 perpendicular to OP, Asymmetry, amount of menton deviation, SNA, angle of lines formed by the S,N, and point A, SNB, angle of lines formed by the S.N, and point B, ANB, angle of lines formed by point A, the N, and point B, SN-PP, angle between the SN plane and the palatal plane; SN-MP, angle between the SN plane and the mandibular plane, U1-SN, angle of the SN plane to the upper incisor; IMPA, angle of the mandibular plane to the lower incisor, T2, 1 day after surgery, T3, 1 month after surgery

^*^p < 0.05/6, significant difference between T2 and T3 by paired *t*-test or Wilcoxon signed rank test

^**^p < 0.001, significant difference between T2 and T3 by paired *t*-test or Wilcoxon signed rank test

^†^*p* < 0.05/6, significant difference between groups by independent *t*-test or Mann-Whiteny *U* test

^‡^*p* < 0.001, significant difference between groups by independent *t*-test or Mann-Whiteny *U* test

### Observation 1 year after surgery

**Fig. 3:**
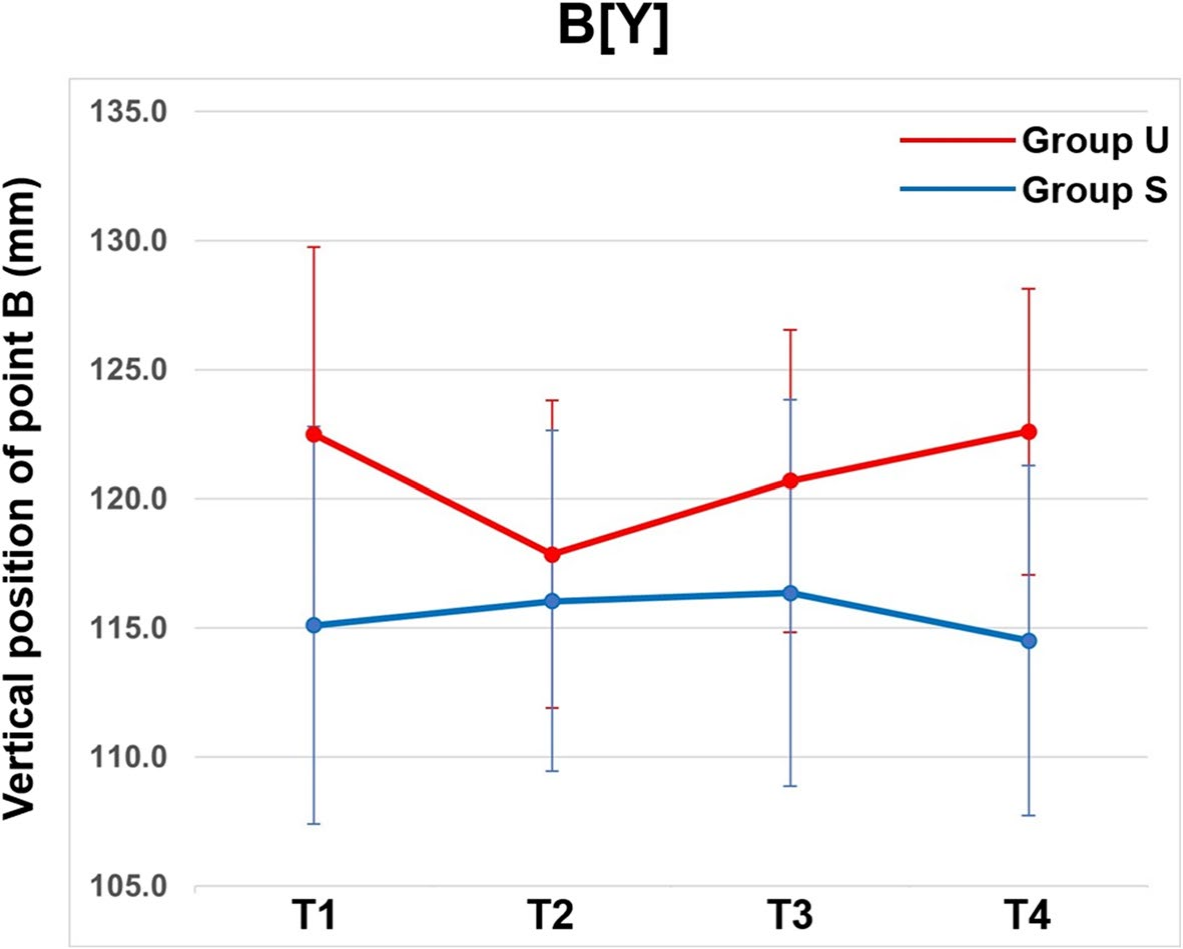
Vertical position of point B in the 2 groups at different time points. B[Y], vertical position of point B; T1, 1 month before surgery; T2, 1 day after surgery; T3, 1 month after surgery; T4, 1 year after surgery

Lastly, at 1 year after surgery, point A moved 0.8 mm (SD, 1.1 mm) posteriorly (*p* < 0.05/6) and 0.5 mm (SD, 1.9 mm) superiorly in group S, while it moved 0.4 mm (SD, 1.4 mm) posteriorly and 0.2 mm (SD, 1.3 mm) inferiorly in group U (Table 5). There were no significant differences in the horizontal and vertical movements of point A 1-year post surgery between the groups. However, point B moved 1.3 mm (SD, 2.5 mm) posteriorly and 1.5 mm (SD, 1.2 mm) superiorly (*p* < 0.001) in group S, while it moved 1.2 mm (SD, 2.7 mm) posteriorly and 4.7 mm (SD, 1.9 mm) inferiorly (*p* < 0.001) in group U (Table 5). The vertical change in point B 1-year post surgery showed statistically significant differences between the two groups (*p* < 0.001), indicating that group U had downward movement of the mandible, compared to the upward movement of the mandible in group S (Fig. 3).

**Table 5:** Comparison of final postoperative changes in cephalometric measurements between groups

T4-T2	Group S	Group U	*p* value
Linear measurements (mm)			
A[X]	-0.8 ± 1.1*	-0.4 ± 1.4	0.471
B[X]	-1.3 ± 2.5	-1.2 ± 2.7	0.879
A[Y]	-0.5 ± 1.9	0.2 ± 1.3	0.284
B[Y]	-1.5 ± 1.2**	4.7 ± 1.9**	0.000‡
U1[Y]	0.3 ± 1.6	1.6 ± 1.8	0.050
U6[Y]	-0.8 ± 1.2*	-0.4 ± 1.7	0.422
OJ	-0.1 ± 1.5	-1.5 ± 2.2	0.049
OB	1.2 ± 1.6*	0.6 ± 1.6	0.385
Menton deviation	0.7 ± 0.5	0.5 ± 0.3	0.227
Angular measurements (º)			
SNA	-0.7 ± 1.2*	-0.8 ± 1.8	0.913
SNB	-0.7 ± 1.3	-0.8 ± 1.6	0.901
ANB	0.0 ± 1.2	0.0 ± 1.2	0.986
SN-PP	0.3 ± 2.1	0.1 ± 1.8	0.771
SN-MP	2.1 ± 2.0**	6.1 ± 3.0**	0.000‡
U1-SN	1.1 ± 5.2	-1.5 ± 5.1	0.193
IMPA	0.6 ± 5.5	-2.1 ± 3.8	0.179

Data are presented as mean ± deviation. Positive and negative values indicate anterior and posterior horizontal changes, respectively; inferior and superior vertical changes, respectively; or increased or decreased dimensional changes, respectively. Group comparisons were tested with independent *t*-test or Mann–Whitney *U* test with Bonferroni correction

*Abbreviations: Group S* stable group, *Group U* unstable group, *A[X]* horizontal position of point A, *B[X]* horizontal position of point B, *A[Y]* vertical position of point A, *B[Y]* vertical position of point B, *U1[Y]* vertical position of upper central incisor, *U6[Y]* vertical position of upper first molar, *OJ* Subtract distance of L1 from U1 parallel to OP, *OB* Subtract distance of L1 from U1 perpendicular to OP, Asymmetry, amount of menton deviation, *SNA* angle of lines formed by the S,N, and point A, *SNB* angle of lines formed by the S.N, and point B; ANB, angle of lines formed by point A, the N, and point B, *SN-PP* angle between the SN plane and the palatal plane, *SN-MP* angle between the SN plane and the mandibular plane; U1-SN, angle of the SN plane to the upper incisor, *IMPA* angle of the mandibular plane to the lower incisor, T2, 1 day after surgery, T4, 1 year after surgery

^*^p < 0.05/6, significant difference between T2 and T4 by paired *t*-test or Wilcoxon signed rank test

^**^p < 0.001, significant difference between T2 and T4 by paired *t*-test or Wilcoxon signed rank test

^†^*p* < 0.05/6, significant difference between groups by independent *t*-test or Mann-Whiteny *U* test

^‡^*p* < 0.001, significant difference between groups by independent *t*-test or Mann-Whiteny *U* test

In both groups, the SN-MP angle increased significantly (*p* < 0.001), and there was a significant difference between the two groups (*p* < 0.001), indicating more clockwise rotation of the mandible in group U.

### Correlation between postoperative vertical movement of mandible and significant variable

At both 1-month and 1-year post surgery, the results of the analysis showed a moderate-to-strong correlation (*r* > 0.40) between postoperative vertical movement of the mandible and B[Y] and OB at initial examination, surgical vertical movement of the B-point, and surgical change of OB and SN-MP angle (Table 6). In terms of Pearson correlation coefficient, the highest correlation was seen between both 1-month (*r* = -0.604, *p* < 0.001) and 1-year (*r* = -0.674, *p* < 0.001) postoperative vertical movement of the mandible and surgical vertical movement of the B-point.

**Table 6:** Correlation between postoperative vertical relapse and variables

	B[Y](T3-T2) (mm)		B[Y](T4-T2) (mm)	
	*r*	*p* value	*r*	*p* value
B[Y] (T1) (mm)	0.544	0.001	0.424	0.013
OB (T1) (mm)	-0.538	0.001	-0.487	0.004
B[Y] (T2 – T1) (mm)	-0.604	< 0.001	-0.674	< 0.001
OB (T2 – T1) (mm)	0.558	0.001	0.440	0.009
SN-MP (T2—T1)	-0.556	0.001	-0.415	0.015
(º)				

*Abbreviations:* B[Y] vertical position of point B, OB, Subtract distance of L1 from U1 perpendicular to OP, A[X] horizontal position of point A, *SN-MP* angle between the SN plane and the mandibular plane, *r*, Pearson correlation coefficient, T1, 1 month before surgery, T2 1 day after surgery, T3 1 month after surgery, T4, 1 year after surgery

**Fig. 4:**
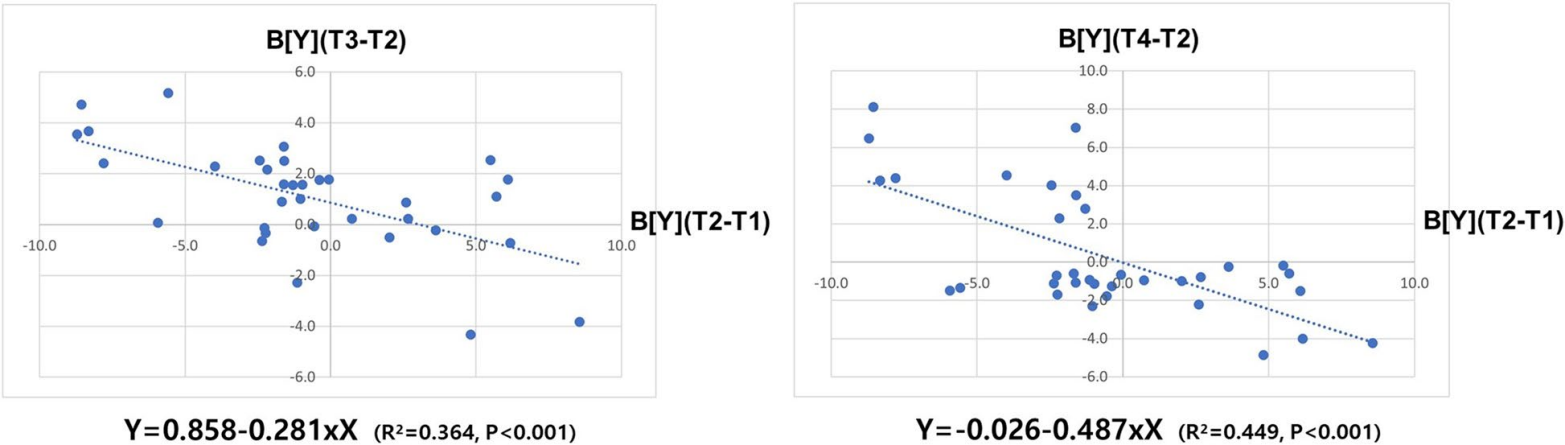
Associations between the amount of vertical change of point B during surgery and the amount of immediate, final postoperative vertical change of point B, respectively. Best-fit lines with determinant coefficient; R^2^ from multiple linear regression analysis. B[Y], vertical position of point B; T1, 1 month before surgery; T2, 1 day after surgery; T3, 1 month after surgery; T4, 1 year after surgery

Five significant variables (B[Y] and OB at initial examination, surgical vertical movement of B-point, and surgical change of OB and SN-MP angle) that showed moderate-to-strong correlation were included in the multiple regression analysis. In the final model, only surgical vertical movement of the B-point was included at both 1-month (R^2^ = 0.364; *p* < 0.001) and 1-year post surgery (R^2^ = 0.449; *p* < 0.001), and showed a linear relationship (Fig. 4).

## Discussion

The aim of this study was to identify factors affecting the unusual downward movement of the mandible exceeding 2 mm 1-year after the surgery-first approach using IVRO, focusing on cephalometric values. Additionally, we determined that a negative correlation exists between the amount of surgical vertical movement of mandible and postoperative vertical movement of mandible. Although the downward movement of the mandible after surgery-first approach with IVRO is uncommon, when it does occur, it can present challenges for orthodontists during the postoperative orthodontic period. Therefore, investigating the factors influencing this unusual downward movement of the mandible after surgery could enhance the predictability of postoperative mandibular changes, providing clinicians with an advantage when planning surgery and postoperative orthodontic treatment.

Previous studies have evaluated the changes occurring after the surgery-first approach using IVRO, with their results confirming its stability even in cases with asymmetry [23, 24] or according to the facial vertical pattern [25]. Choi et al. [12, 13] reported that 1 year after surgery, the vertical movement of the B-point was -1.9 ± 2.0 mm; Kim et al. [15] reported a corresponding value of -2.86 ± 1.39 mm, while Jeong et al. [16] reported a corresponding value of -0.6 ± 1.0 mm. Immediately after the surgery-first approach with IVRO, which does not implement rigid fixation between the proximal and distal segments, the distal segment may rotate clockwise and the mandible may move downward. However, once the mandible achieves union between segments and premature occlusal contacts are eliminated during postoperative orthodontic treatment, along with rehabilitation of the pterygomasseteric sling, a predictable upward movement of the mandible of approximately 0–2 mm is observed, which leads to a stable outcome [12, 13]. While almost all patients followed this predictable movement pattern, there were very rare cases in which patients exhibited an unusual downward movement of mandible from immediately after the surgery up to 1 year post-operatively, thus resulting in a clinical presentation of anterior openbite. These instances required additional efforts from the orthodontist to stabilize the post-surgical occlusion. Despite representing a small minority of cases, these unusual movements posed significant stressors for the orthodontist. Identifying the characteristics of these patients and defining at-risk groups could allow for more predictable outcomes of the surgery-first approach with IVRO. Therefore, the present study intended to identify factors influencing the unusual downward movement of the mandible 1 year after the surgery-first approach with IVRO, with the ultimate aim of enhancing the predictability. Traditionally, any movement exceeding 2 mm post-surgical healing is considered to be significant [20]; therefore, this study labeled patients who showed more than 2 mm of mandibular downward movement 1-year post-surgery as vertically unstable and evaluated the factors affecting this group. We found that the surgical vertical movement of the B-point is the most statistically important factor affecting the 1-year postoperative downward movement of the B-point after the surgery-first approach with IVRO.

At the initial examination, Group U showed a shallower anterior overbite with openbite of 1.6 mm. An anterior openbite can occur as a result of vertical skeletal patterns of either posterior downward rotation of the maxilla or clockwise rotation of the mandible [26]. However, there were no significant differences in the SN-PP and SN-MP angles between the groups at the initial examination, confirming that there were no differences in vertical facial patterns such as dolichofacial or brachyfacial between the groups. Further, although not statistically significant, it was observed that the vertical value of the mandibular B-point was greater by 7.4 mm in Group U, indicating a longer anterior facial height pre-surgery.

One day after the surgery, the amount of posterior superior movement of the maxilla was approximately the same for both groups; however, in Group U, the vertical movement of the mandible was significantly greater by 5.5 mm upward. Although not statistically significant, the anterior overbite in Group U was increased 2.2 mm greater than that in Group S. The reason for the greater surgical changes in vertical position of B-point and anterior overbite in Group U is that, at the initial examination, Group U had higher B[Y] values and lower OB values, thus necessitating greater surgical correction. Further, while not statistically significant, the mandible in Group S rotated more clockwise compared to Group U, ultimately resulting in a larger increase in the SN-MP value.

One month after surgery, the mandible's B-point in Group U showed a significant downward movement of 2.8 mm compared to immediately post-surgery. Although not statistically significant, Group U exhibited more clockwise rotation of the mandible, while the anterior overjet decreased in a manner that was different than that shown in Group S. This pattern persisted until 1 year after the surgery, at which point it was confirmed that the B-point of the mandible in Group U had moved downward by 4.7 mm, the mandible had rotated clockwise by 6.1º and the anterior overjet had decreased by 1.5 mm compared to 1-day post-surgery. Choi et al. [12, 13], Ahn et al. [14], and Jeong et al. [16] reported that after IVRO, mandibular clockwise rotation occurs; however, in the case of a surgery-first approach, compensatory counterclockwise rotation of the mandible, particularly the distal segment, occurs as premature occlusal contact disappears, and the masticatory muscles recover during the postoperative orthodontic period [12, 13]. As a result, primarily upward movement of the mandible is observed compared to the immediate postoperative state. In the case of Group U in this study, significant clockwise rotation of the mandible occurred along with a downward movement of more than 2 mm 1 year after surgery, despite the surgery-first approach. Additionally, the anterior overjet showed a decreasing trend in Group U during the postoperative period, suggesting that the center of rotation of the mandible is located between the central and anterior parts of the mandible body. Nihara et al. [18] discussed the adaptive rotation of the mandible following IVRO due to the pulling force of the masticatory muscles but found it difficult to define a specific point for the center of adaptive rotation, as it is broadly located between the coronoid process and the menton. In the current study, the patients in Group U showed significant upward movement of the mandible during surgery, and the subsequent considerable downward movement during the postoperative period affected the shifting of the center of rotation forward, which likely contributed to the reduction in anterior overjet during clockwise rotation. Efforts to resolve this decreased overjet during the postoperative orthodontic treatment were evident from the decrease in IMPA in Group U 1-year post-surgery, although this was not statistically significant.

One month and 1 year postoperatively, five variables (B[Y] and OB at initial examination, surgical vertical movement of B-point, and surgical change of OB and SN-MP angle) demonstrated a moderate-to-strong correlation with the downward movement of the mandible's B-point, but the results of multiple regression analysis revealed that only the surgical vertical movement of B-point exhibited a significant association and a linear relationship. Similarly, Choi et al. [12] confirmed that an extensive upward movement of B-point during surgery is associated with a considerable postoperative downward movement of B-point 1 year later. Clinically, a vertical downward movement exceeding 2 mm is considered significant. Based on the regression equations derived from the multiple regression analysis (Fig. 4. 1-month post surgery: Y = 0.858–0.281xX, 1-year post surgery: Y = -0.026–0.487xX), this study suggests that, when the surgical upward movement of B-point exceeds 4 mm, vertical downward movement should be anticipated and monitored carefully. In practice, while the orthodontist faced challenges during postoperative orthodontic treatment in Group U, the use of TADs expanded the scope of orthodontic treatment, and the final treatment outcomes of Group U were comparable to those of patients in Group S. However, if a surgery-first approach using IVRO anticipates an upward movement of the mandible exceeding 4 mm for correction of a longer anterior facial height or shallow anterior overbite at initial examination, it is prudent to prepare for an unusual downward movement of mandible. Orthodontists should be prepared for this possibility, and it may be advisable to inform patients about the potential for a prolonged postoperative orthodontic period and the possibility of additional genioplasty.

This study is the first to identify the factors affecting the unusual downward movement of the mandible 1-year after the surgery-first approach using IVRO, and it has a specific focus on cephalometric values. The limitations of this study include its retrospective nature and the short duration of the postoperative observation period. Additionally, despite the possibility that genioplasty could influence the adaptive process of the mandible postoperatively, this study included both patients who underwent genioplasty and those who did not, thus not excluding the effect of genioplasty on mandibular postoperative adaptive process. Although there are studies reporting stable outcomes when the surgery-first approach is performed on patients with facial asymmetry [23, 24], these studies involved SSRO. In contrast, this study utilized IVRO, and therefore, the potential impact of facial asymmetry on postoperative stability was not considered, which is a limitation of this study. Moreover, despite calculating the sample size and focusing on very rare cases with unusual outcomes, the number of samples remains small. In future research, it would be beneficial to increase the number of samples if similar results are observed in other patients, and it would also be useful to conduct studies that include longer-term follow-up periods.

## Conclusions

Almost all patients who underwent the surgery-first approach using IVRO exhibited the anticipated upward movement of the mandible 1 year postoperatively, resulting in a stable outcome. However, a very small number of patients showed downward movement of the mandible up to 1-year post-surgery, which posed challenges for orthodontists in stabilizing the occlusion during the postoperative orthodontic period. The findings in this study revealed that such unusual downward movement of the mandible occurred with mandibular clockwise rotation and decrease of anterior overjet after a surgery-first approach using IVRO and is correlated with the amount of upward movement of mandible during the surgery. If a significant upward movement of the mandible is anticipated while planning surgery, orthodontists should prepare for the possibility of subsequent unusual anterior openbite along with a tendency for the anterior overjet to decrease during the postoperative orthodontic period. Also, it may be advisable to inform patients about the potential for a prolonged postoperative orthodontic period and the possibility of additional genioplasty.

## Data Availability

No datasets were generated or analysed during the current study.

## Abbreviations

TADs: Temporary Anchorage Devices
IVRO: Intraoral Vertical Ramus Osteotomy
IMF: Intermaxillary Fixation
PT: Physiotherapy
T1: Before surgery
T2: 1 Day before surgery
T3: 1 Month after surgery
T4: 1 Year after surgery
S: Sella
N: Nasion
U1: Tip of upper central incisor
U6: Tip of distal cusp of upper first molar
OJ: Anterior overjet
OB: Anterior overbite
SN-PP: Angle between the SN plane and the palatal plane
SN-MP: Angle between the SN plane and the mandibular plane
IMPA: Angle of the mandibular plane to the lower incisor
